# Associations of healthy lifestyles with cerebrospinal fluid biomarkers of Alzheimer’s disease pathology in cognitively intact older adults: the CABLE study

**DOI:** 10.1186/s13195-021-00822-7

**Published:** 2021-04-19

**Authors:** Xiao-He Hou, Wei Xu, Yan-Lin Bi, Xue-Ning Shen, Ya-Hui Ma, Qiang Dong, Lan Tan, Jin-Tai Yu

**Affiliations:** 1 Department of Neurology, Qingdao Municipal Hospital, Qingdao University, Qingdao, China; 2 Department of Anesthesiology, Qingdao Municipal Hospital, Qingdao University, Qingdao, China; 3 Department of Neurology and Institute of Neurology, Huashan Hospital, Shanghai Medical College, Fudan University, 12th Wulumuqi Zhong Road, Shanghai, 200040 China

**Keywords:** Alzheimer’s disease, Lifestyle, Social isolation, Physical activity, Cerebrospinal fluid, Biomarker

## Abstract

**Objective:**

We aimed to investigate the associations between healthy lifestyles and Alzheimer’s disease (AD) biomarkers in cerebrospinal fluid (CSF).

**Methods:**

A total of 1108 cognitively intact individuals from Chinese Alzheimer’s Biomarker and LifestylE (CABLE) study were examined to evaluate the associations of AD biomarkers with healthy lifestyle factors, including no current smoking, no harmful drinking, absence of social isolation, and regular physical activity. The participants were categorized into groups of favorable, intermediate, and unfavorable lifestyles according to the lifestyle factors. The associations between overall lifestyle and CSF biomarkers were also analyzed.

**Results:**

Among cognitively intact older adults, those having more social engagement had lower CSF tau (*p* = 0.009) and p-tau (*p* < 0.001) than those who had social isolation. Regular physical activity was associated with higher CSF Aβ42 (*p* = 0.013) and lower levels of CSF tau (*p* = 0.036) and p-tau (*p* = 0.007). However, no significant associations were found of smoking status or alcohol intake with CSF biomarkers. When the overall lifestyle of the participants was evaluated by all the four lifestyle factors, favorable lifestyle profiles were related to lower levels of CSF tau (*p* < 0.001) and p-tau (*p* < 0.001).

**Conclusions:**

These findings suggest that healthy lifestyles had a beneficial effect on AD pathology among cognitively intact elders.

**Supplementary Information:**

The online version contains supplementary material available at 10.1186/s13195-021-00822-7.

## Introduction

Alzheimer’s disease (AD) is the most common cause of dementia, which becomes a great burden on patients and society [1]. AD risk can be influenced by both genetic and environmental factors. Recent analyses have shown that modifiable lifestyle factors might also influence the risk of AD [2]. It has been found that several healthy lifestyle factors are associated with lower risk of dementia [3–8]. And dementia risk can be lowered by the combination of favorable lifestyle factors [9, 10]. In 2019, WHO published a guideline for the prevention of cognitive decline and dementia, which included tobacco cessation interventions, physical activity interventions, social activity interventions, and interventions for alcohol use disorders [11]. Estimates suggest that over a third of dementia cases could be prevented if currently established modifiable risk factors were eliminated at a population level [12].

Cerebrospinal fluid (CSF) β-amyloid 1-42 (Aβ42), total tau, and phosphorylated tau (p-tau) are validated biomarkers for AD. AD biomarkers may become abnormal more than 20 years before the diagnosis of dementia [13]. In the newly published NIA-AA research framework, the levels of CSF Aβ42, tau and p-tau have been established as core AD biomarkers to define AD biologically [14]. There were some previous studies on the associations between several healthy lifestyle factors and AD biomarkers [15–17] which yielded inconsistent results. Whether lifestyle factors have an effect on AD core pathologies is less known.

We aimed to investigate the associations of common healthy lifestyles including no current smoking, no harmful drinking, absence of social isolation, and regular physical activity with CSF biomarkers of Alzheimer’s disease pathology in a large sample of 1108 cognitively intact individuals.

## Materials and methods

### Study participants

A total of 1108 cognitively normal individuals were included from the Chinese Alzheimer’s Biomarker and LifestylE (CABLE) study [18]. CABLE is an ongoing large-scale study mainly focusing on Alzheimer’s risk factors and biomarkers in Chinese Han population. Individuals were recruited at Qingdao Municipal Hospital, Shandong Province, China. All enrolled participants were Han Chinese aged between 40 to 90 years. The exclusion criteria include the following: (1) central nervous system infection, head trauma, epilepsy, multiple sclerosis, or other major neurological disorders; (2) major psychological disorders; (3) severe systemic diseases (e.g., malignant tumors); and (4) family history of genetic diseases other than AD. Demographic information and medical history were collected by an electronic medical record system. General cognitive function of participants was assessed by China-Modified Mini-Mental State Examination (CM-MMSE) and Montreal Cognitive Assessment. The cutoff values of MMSE were 19 for illiterate individuals, 22 for individuals with 1 to 6 years of education, and 26 for individuals with 7 or more years of education. The cutoff values of MoCA were 14 for illiterate individuals, 20 for individuals with 1 to 6 years of education, and 25 for individuals with 7 or more years of education in the screening of MCI [19].

### CSF AD biomarker measurements and APOE genotyping

Cerebrospinal fluid of the participants was collected by lumbar puncture in 10 ml polypropylene tubes before being sent to the lab within 2 h. CSF samples were centrifuged at 2000*g* for 10 min. The thaw/freezing cycle was limited not to surpass 2 times. Baseline CSF Aβ_1–42_, Aβ_1–40_, tau, and p-tau181 were determined with the ELISA kit (Innotest β-AMYLOID (1-42), β-AMYLOID (1-40), hTAU-Ag, and PHOSPHO-TAU (181p); Fujirebio, Ghent, Belgium) on the microplate reader (Thermo Scientific™ Multiskan™ MK3). In quality control, all CSF samples were measured in duplicate. Duplicate measures which had a coefficient of variation ≥ 15% were excluded. Besides, extreme values which were 4-fold SD greater or smaller than the mean value were also removed from the analysis. Data were excluded in quality control for Aβ42 (*n* = 162, 14.6%), CSF Aβ40 (*n* = 198, 17.9%), CSF tau (*n* = 92, 8.3%), and CSF p-tau (*n* = 90, 8.1%).

DNA was extracted from the blood samples using QIAamp DNA Blood Mini Kit (250) and amplified by the polymerase chain reaction. APOE alleles defined by rs7421 and rs429358 were genotyped by restriction fragment length polymorphism (RFLP) technology.

### Lifestyle assessment

Lifestyle factors implicated in the risk of dementia were assessed by a comprehensive questionnaire, including (1) smoking status, (2) alcohol consumption, (3) social engagement, and (4) physical activity [11]. Each lifestyle factor was divided into two categories (healthy and unhealthy). Smoking status was categorized as current smoking or no current smoking. Alcohol consumption was categorized into harmful drinking group (pure alcohol > 25 g/d for men and > 15 g/d for women) and no or moderate drinking group according to the dietary guidelines for Chinese residents. Social engagement was categorized into social isolation and absence of social isolation according to the living arrangements (living alone, with spouse or with others) and close relationship with relatives, friends, and neighbors. If a participant was living alone or had no close relationship with relatives, friends, and neighbors, then we considered him or her as being socially isolated. As for physical activity, we assessed the frequency of physical activities and categorized into regular physical activity and lack of physical activity. Regular physical activity was regarded as a healthy lifestyle which was defined as moderate physical activity every day. Detailed questionnaire was provided in Additional file 1: Questionnaire used in the evaluation of healthy lifestyles.

### Statistical analysis

The associations between healthy lifestyle factors and AD biomarkers were analyzed using multivariable linear regression. Age, gender, educational level, and APOE ε4 status were included as covariates. The values of CSF AD biomarkers were transformed to achieve or approximate a normal distribution (Kolmogorov-Smirnov test *P* > 0.05) via “car” package of R software in case of skewed distribution. In subgroup analysis stratified by APOE ε4 status, only age, gender, and educational level were included as covariates. The lifestyle of each participant was evaluated based on the assessments of the four lifestyle factors. In addition to consideration of their categories on each individual lifestyle variable, the lifestyle categories were defined by the number of healthy lifestyle factors the participants adhere to. The participants were categorized into favorable lifestyle group (4 healthy lifestyle factors), intermediate lifestyle group (3 healthy lifestyle factors), and unfavorable lifestyle group (≤ 2 healthy lifestyle factors). The associations between lifestyle categories and AD biomarkers were also analyzed using multivariable linear regression. Statistical analysis was carried out using R version 3.5.3.

## Results

### Demographic and clinical characteristics of included participants

A total of 1108 participants were enrolled in this study. The 1108 participants included 461 female participants (41.6%). The baseline characteristics of the participants are summarized in Table 1. In brief, the mean age of the included participants was 61.1 ± 11.0 years old. All the participants were cognitively unimpaired with a mean CM-MMSE score of 28.2 ± 1.9 (range 19 to 30). The mean MoCA score of the participants was 23.8 ± 4.0 (range 14 to 30).

**Table 1:** Characteristics of study subjects

Characteristic	No. (*n* = 1108)
Age (years)	61.1 ± 11.0 (41 to 89)
Sex (F/M)	461/647
Education (years)	9.8 ± 4.4
CM-MMSE	28.2 ± 1.9
MoCA	23.8 ± 4.0
^*^APOE ε4 (carriers/non-carriers)	161/800
^*^CSF Aβ42 (pg/ml)	167.6 ± 79.5
^*^CSF Aβ40 (pg/ml)	5981.8 ± 2728.5
^*^CSF tau (pg/ml)	171.3 ± 76.1
^*^CSF p-tau (pg/ml)	36.8 ± 9.1
Healthy lifestyle factors (*n*, %)	
No current smoking	888 (80.1%)
No or moderate drinking	951 (85.8%)
Regular physical activity	275 (24.8%)
Absence of social isolation	868 (78.3%)
No. of healthy lifestyle factors (*n*, %)	
0	9 (0.8%)
1	84 (7.6%)
2	302 (27.3%)
3	558 (50.4%)
4	155 (14.0%)

Summarized as mean ± standard deviation (SD) for continuous data and count for categorical data. *Abbreviations*: CSF, cerebrospinal fluid; MMSE, Mini-Mental State Examination; p-tau, phosphorylated tau

* Data were missing for CSF Aβ42 (*n* = 162, 14.6%), CSF Aβ40 (*n* = 198, 17.9%), CSF tau (*n* = 92, 8.3%), CSF p-tau (*n* = 90, 8.1%), and APOE ε4 statues (*n* = 147, 13.3%)

### Associations between healthy lifestyle factors and CSF AD biomarkers

**Fig. 1:**
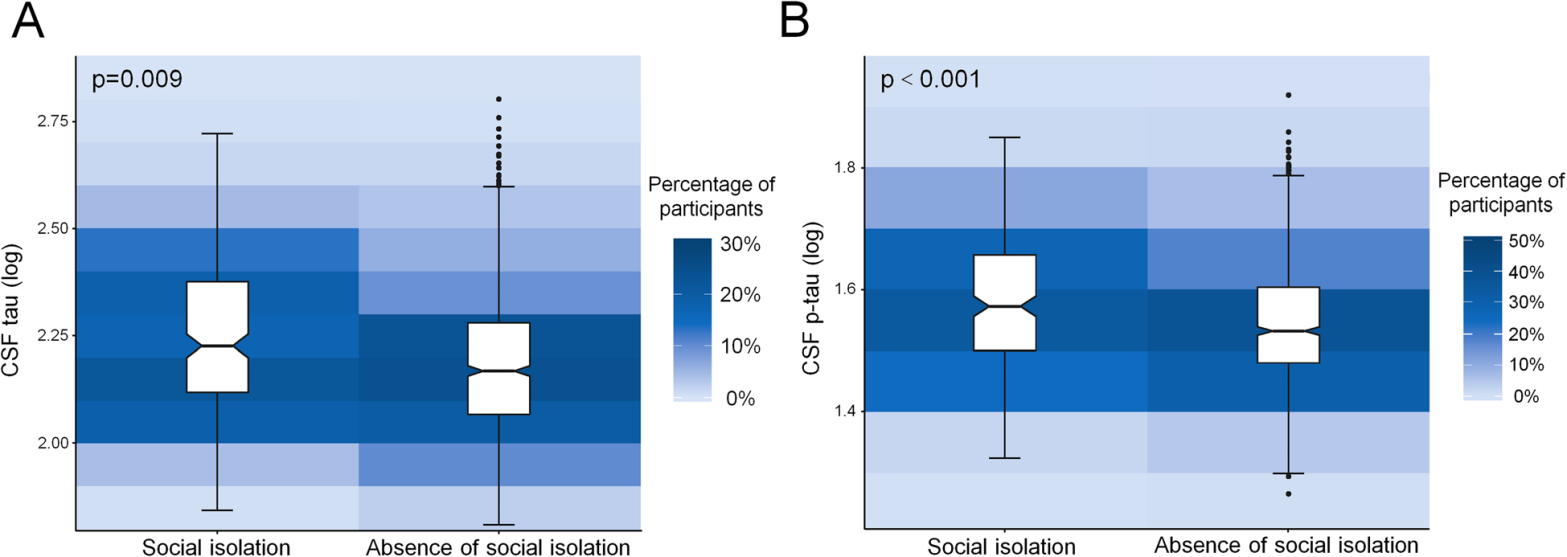
Associations of social isolation with CSF tau and p-tau levels. **a** Participants without social isolation had lower CSF tau levels compared to those with social isolation (*p* = 0.009). **b** Participants without social isolation had lower CSF p-tau levels compared to those with social isolation (*p* < 0.001)

**Fig. 2:**
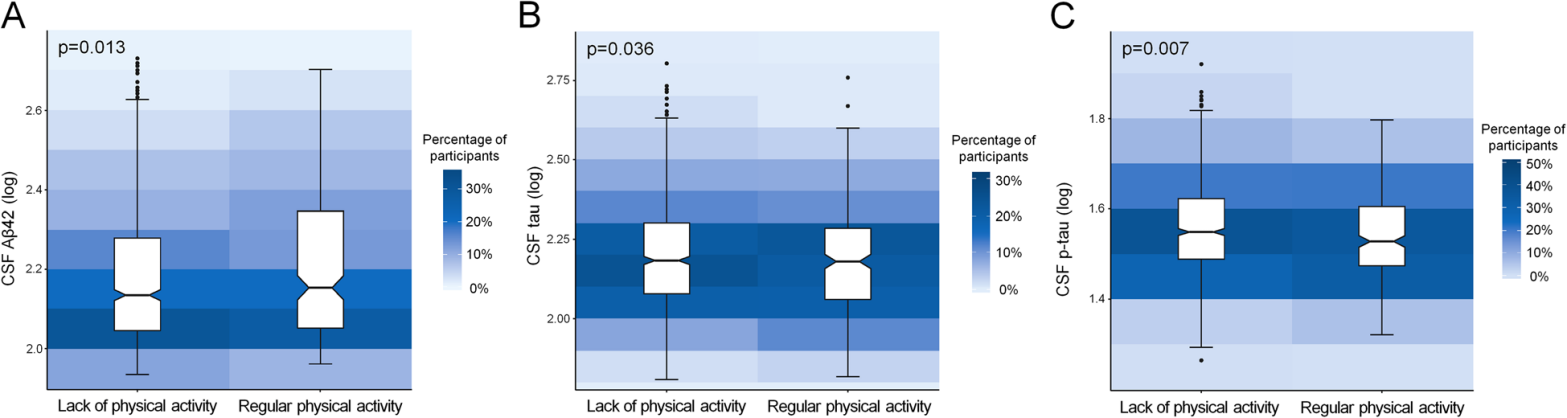
Associations of physical activity with CSF Aβ42 and p-tau levels. Participants who were physically active had higher CSF Aβ42 levels (**a**), as well as lower CSF tau (**b**) and p-tau levels (**c**) compared to those who had a lack of regular physical activity

The results are presented in Table 2. As for the factor of social engagement, after adjusting for age, sex, educational level, and APOE ε4 status, participants without social isolation showed lower levels of CSF tau (*p* = 0.009) and p-tau (*p* < 0.001) than those with social isolation (Fig. 1). Besides, participants engaging in regular physical activity had higher CSF Aβ42 (*p* = 0.013), as well as lower levels of CSF tau (*p* = 0.036) and p-tau (*p* = 0.007) compared to those with a lack of regular physical activity (Fig. 2). However, no significant associations were found of smoking status and alcohol intake with CSF biomarkers.

**Table 2:** Association between lifestyle factors and AD biomarkers

	Current smoking		P^*^	*P*^#^
	Yes (*n* = 220)	No (*n* = 888)		
CSF Aβ42	162.25 ± 71.75	169.01 ± 81.40	0.47	0.17
CSF tau	170.81 ± 70.35	171.43 ± 77.54	0.78	0.29
CSF p-tau	36.57 ± 8.57	36.89 ± 9.23	0.83	0.47
	Alcohol consumption			
	Harmful drinking (*n* = 157)	No or moderate drinking (*n* = 951)		
CSF Aβ42	178.53 ± 94.72	165.82 ± 76.65	0.35	0.44
CSF tau	175.84 ± 80.03	170.55 ± 75.48	0.63	0.12
CSF p-tau	37.11 ± 10.00	36.79 ± 8.95	0.97	0.35
	Absence of social isolation			
	No (*n* = 240)	Yes (*n* = 868)		
CSF Aβ42	175.89 ± 89.94	165.33 ± 76.30	0.27	0.19
CSF tau	188.17 ± 76.40	166.67 ± 75.44	< 0.01	< 0.01
CSF p-tau	39.11 ± 9.09	36.21 ± 9.01	< 0.01	< 0.01
	Regular physical activity			
	No (*n* = 833)	Yes (*n* = 275)		
CSF Aβ42	163.96 ± 76.65	178.96 ± 87.03	0.03	0.01
CSF tau	173.73 ± 78.20	164.03 ± 69.15	0.10	0.04
CSF p-tau	37.25 ± 9.32	35.57 ± 8.28	0.01	< 0.01

*Unadjusted

^#^Adjusted for age, sex, educational level, and APOE ε4 status

It has been reported that CSF Aβ42/Aβ40, tau/Aβ42, and p-tau/Aβ42 ratios are better predictors of brain amyloid deposition than themselves alone [20, 21]. Therefore, we also explored the associations between the four lifestyle factors and AD biomarkers. Only regular physical activity was found to be associated with lower CSF tau/Aβ42 (*p* < 0.001) and p-tau/Aβ42 ratios (*p* < 0.001, Additional file 1: Supplementary Fig. 1). We did not find any association between other three healthy lifestyle factors and the ratios of AD biomarkers.

### Associations between healthy lifestyle categories and CSF AD biomarkers

We then categorized the participants into favorable lifestyle group (4 healthy lifestyle factors), intermediate lifestyle group (3 healthy lifestyle factors) and unfavorable lifestyle group (≤ 2 healthy lifestyle factors). The CM-MMSE and MoCA scores were not significantly different among the three groups (*p* = 0.38 for CM-MMSE and *p* = 0.22 for MoCA). The levels of CSF tau and p-tau were found to be reduced across lifestyle categories from unfavorable lifestyle group to favorable lifestyle group (*p* < 0.01 for CSF tau and p-tau, Additional file 1: Supplementary Table 1). CSF Aβ42 levels were not significantly different among participants with different lifestyle profiles (*p* = 0.55).

**Fig. 3:**
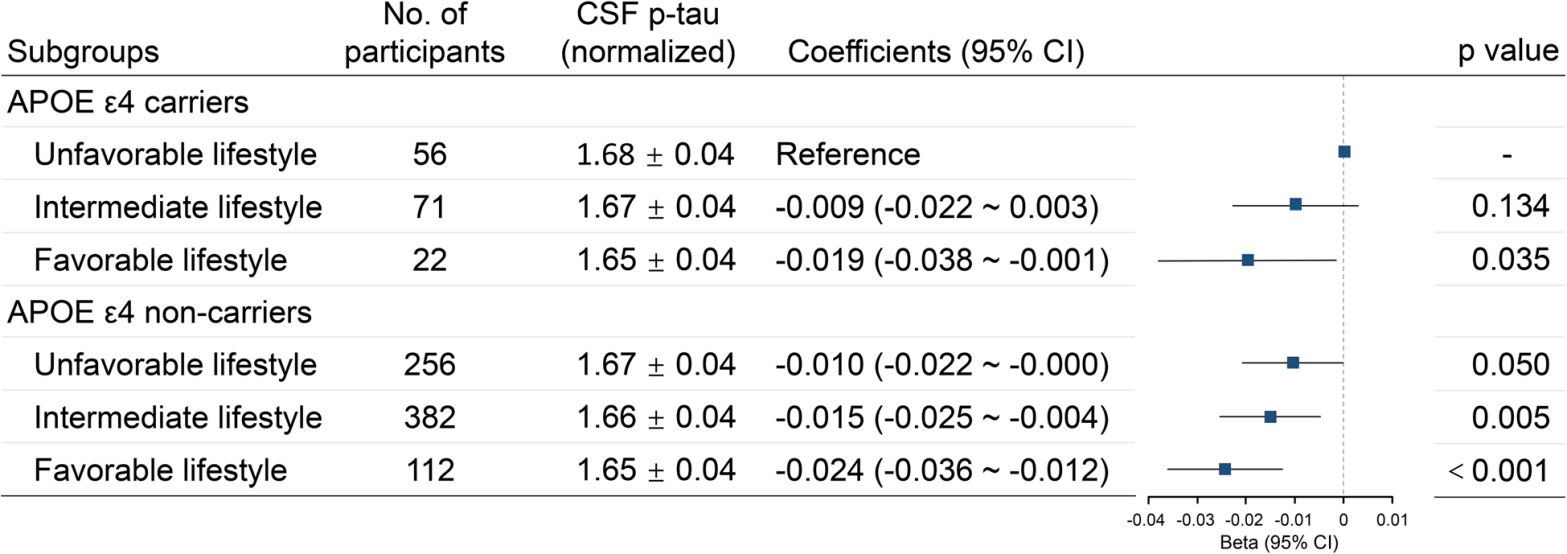
CSF p-tau levels in subgroups stratified by APOE ε4 status and lifestyle categories. Lower genetic risk (APOE ε4 negative) and more favorable lifestyles were both associated with lower levels of CSF p-tau. A downtrend of CSF p-tau was revealed with decreased genetic risk (APOE ε4 status) and an increasingly healthy lifestyle

When the participants were divided into APOE ε4 carriers and APOE ε4 non-carriers, the associations of lifestyle categories with CSF tau and p-tau were found in both APOE ε4 carriers and APOE ε4 non-carriers (Additional file 1: Supplementary Table 1). In our study, APOE ε4 status was also found to be associated with CSF p-tau levels. APOE ε4 carriers had higher CSF p-tau levels than APOE ε4 non-carriers (*p* = 0.015). Therefore, we classified the participants into six groups according to their APOE ε4 status and lifestyle categories. When APOE ε4 status and lifestyle categories were combined, both of low genetic risk (APOE ε4 negative) and favorable lifestyle were associated with decreased CSF p-tau levels (Fig. 3).

The associations of lifestyle categories with CSF Aβ42/Aβ40, tau/Aβ42, and p-tau/Aβ42 ratios were also analyzed. CSF tau/Aβ42 and p-tau/Aβ42 ratios were found to be reduced across lifestyle categories in all participants as well as in both APOE subgroups (Additional file 1: Supplementary Table 2).

We then only included regular physical activity and social engagement in the analysis because smoking status and harmful alcohol intake were not independently associated with CSF AD biomarkers. The participants were classified into three groups according to the number of the two healthy lifestyles participants adhere to (having both lifestyles, having one lifestyle and having no healthy lifestyle). As expected, participants with more healthy lifestyles had lower CSF tau and p-tau levels (*p* < 0.01 for CSF tau and p-tau, Additional file 1: Supplementary Table 3). These results indicated that regular physical activity and social engagement could significantly influence AD biomarkers. In addition, we also performed an interaction analysis. An interaction was found between regular physical activity and social engagement in the influence of CSF p-tau (*p* = 0.04, Additional file 1: Supplementary Table 4).

## Discussion

In the present study, we revealed beneficial effects of social engagement and physical activity on AD pathology. Individuals who had social engagement had significantly lower levels of CSF tau and p-tau than those who had social isolation. Regular physical activity was associated with higher levels of CSF Aβ42, as well as lower levels of CSF tau and p-tau. These results are consistent with previous studies demonstrating beneficial effects of social engagement and physical activity on AD risk and cognitive function [6, 22, 23]. In addition, favorable lifestyle profiles evaluated by the four healthy lifestyles were related to lower CSF tau (*p* < 0.001) and p-tau (*p* < 0.001) levels.

Although social engagement is an important factor associated with AD risk, the associations of social engagement with AD biomarkers have rarely been reported. Donovan et al. showed that individuals who had greater feelings of loneliness had more cortical amyloid burden in a cross-sectional study [24]. But the authors indicated that loneliness might be a symptom rather than a cause of amyloid accumulation. Reijis et al. investigated the effects of later life lifestyle factors on AD biomarkers using a cohort of SCD and MCI patients. But they found no association between social activity and AD biomarkers [15]. In the current study, we found that social engagement, which was evaluated by living arrangements and self-reported intimate relationships, was associated with lower levels of CSF tau and p-tau. This association has not been reported before. It has been shown that brain-derived neurotrophic factor (BDNF) levels are increased in individuals with more social support [25]. BDNF was found to have protective effects against tau-related neurodegeneration [26]. Social engagement may protect against adverse changes in AD biomarkers by increasing the production of BDNF. Further research is required on the mechanisms of social engagement in AD pathology.

More studies focused on physical exercise than social activity and demonstrated physical exercise could influence AD biomarkers. The effects of physical activity on AD biomarkers were revealed in animal models of AD [27, 28]. Accumulating evidence from cohort and cross-sectional studies with human participants has suggested that physical activity is beneficial to promoting a favorable profile of AD biomarkers in humans [16, 29–32]. It is worth noting that moderate but not vigorous physical activity had beneficial effects on dementia [31, 33]. The participants included in our study were older adults. Most of the participants did not have vigorous physical activity in their daily life. With a large sample size, our study further confirmed the associations of physical activity with decreased Aβ42 and tau burden. In addition to CSF tau and p-tau, physical activity also had an effect on CSF Aβ42, tau/Aβ42, and p-tau/Aβ42 ratios, which is different from social engagement. Several possible mechanisms regarding how physical activity is involved in AD pathology have been proposed. Physical activity could modulate selective biochemical changes [34], including the levels of high density lipoprotein, triglyceride and insulin, all of which may play a role in the production and/or clearance of Aβ [35]. These might be one of the reasons why physical activity could influence CSF Aβ levels. Besides, physical activity may protect against AD pathology by increasing cerebral blood flow or the production of BDNF [36].

Smoking and alcohol intake have been associated with AD as well [3]. Cerebral oxidative stress is a potential mechanism in which smoking elevates AD risk. It has been reported that oxidative stress could promote abnormal tau phosphorylation in the brain [37], but the association between CSF p-tau and smoking has not been reported. We did not find any difference in the levels of Aβ42, t tau, or p tau between current smoking group and no current smoking group in this study, which suggested that smoking might contribute to AD risk by brain oxidative damage rather than promoting AD pathology. Kok et al. reported an association between beer drinking and decreased Aβ immunoreactivity in the brain [38]. But they did not find any association between the amount of alcohol consumed and Aβ aggregation. A dose–response association between alcohol intake and the risk of dementia has been reported [39]. Light-to-moderate alcohol consumption could lower the risk of dementia. However, it is not recommended for individuals having no alcohol consumption to start drinking in consideration of other health risks in WHO guidelines. Therefore, we considered no harmful drinking rather than moderate drinking as a healthy lifestyle. Similar to smoking, no significant associations were found of alcohol intake with CSF biomarkers.

The overall effect of the combination of lifestyle factors on AD biomarkers has not been reported. In our study, we categorized the participants into three groups (favorable, intermediate, and unfavorable lifestyle groups) according to the number of healthy lifestyle factors the participants adhered to. Consistent with previous studies showing that favorable lifestyles were associated with AD risk [9, 10], our study revealed the associations between healthy lifestyles and AD biomarkers including CSF tau and p-tau. Besides, more favorable lifestyles could reduce CSF tau and p-tau levels in both APOE ε4 carriers and non-carriers. The results indicated that adhering to a healthy lifestyle was a potential way to prevent AD in early stage in spite of APOE ε4 status. In the analysis only using regular physical activity and social engagement in the evaluation of healthy lifestyles, we also found significant associations of healthy lifestyles with CSF tau and p-tau. These results suggested that physical activity and social engagement might be the main factors which could influence AD biomarkers. We also found an interaction between regular physical activity and social engagement, which indicated that participation in physical activity and social engagement at the same time might have better effects on AD prevention.

The mean educational level of the participants was 9.8 years in our study, which was lower than those of many other cohorts such as ADNI. Because some of the elders in China did not have chance to receive formal education when they were young, individuals with a high educational level tend to have a healthy lifestyle. We also found a positive association between years of education and healthy lifestyles. And only 14% of the participants had a favorable lifestyle in our study. Further studies are still needed to investigate the associations between lifestyles and biomarkers with more participants having a favorable lifestyle.

## Limitations

There are some limitations in this study. The lifestyle factors were self-reported in our study. Physical activity was not measured objectively. Besides, we lacked validated scales to measure social engagement. In addition, the cross-sectional studies can reveal the associations between lifestyles and AD biomarkers, but cannot prove causal relationships. The follow-up study of CABLE participants has already been initiated. The causal relationships between lifestyle and AD biomarkers will be investigated in our future studies.

## Conclusions

In conclusion, our present study revealed the associations of social isolation and physical activity with cerebrospinal fluid biomarkers of Alzheimer’s disease pathology in cognitively intact older adults. These results provided evidence for lifestyle interventions in AD prevention and strengthened the current recommendations from WHO on AD prevention.

## Supplementary Information

**Additional file 1: Supplementary Fig. 1.** Associations of physical activity with CSF tau/Aβ42 and p-tau/ Aβ42 ratios. (A) Participants who were physically active had lower CSF tau/Aβ42 compared to those who had a lack of physical activity (*p* < 0.001). (B) Participants who were physically active had lower p-tau/Aβ42 levels compared to those who had a lack of physical activity (*p* < 0.001). **Supplementary Table 1**. Association between lifestyle categories and CSF AD biomarkers; **Supplementary Table 2.** Association of lifestyle categories with CSF Aβ42/Aβ40, tau/Aβ42 and p-tau/Aβ42 ratios; **Supplementary Table 3.** Association between lifestyle categories (defined by physical activity and social engagement) and CSF AD biomarkers. **Supplementary Table 4.** Interaction analysis between physical activity and social engagement. Questionnaire used in the evaluation of healthy lifestyles.

## Data Availability

The datasets used and/or analyzed during the current study are available from the corresponding author on reasonable request.

## Abbreviations

Aβ42: β-amyloid 1-42
AD: Alzheimer’s disease
CABLE: Chinese Alzheimer’s Biomarker and LifestylE
CSF: Cerebrospinal fluid
CM-MMSE: China-Modified Mini-Mental State Examination
p-tau: Phosphorylated tau
