# Supplementary material for: Associations of healthy lifestyles with cerebrospinal fluid biomarkers of Alzheimer’s disease pathology in cognitively intact older adults: the CABLE study
Source: Alzheimers Res Ther. 2021 Apr 19;13:81. doi: 10.1186/s13195-021-00822-7 (PMC8056495; doi:10.1186/s13195-021-00822-7)
Supplement: Supplementary file 1 — Additional file 1: Supplementary Fig. 1. Associations of physical activity with CSF tau/Aβ42 and p-tau/ Aβ42 ratios. (A) Participants who were physically active had lower CSF tau/Aβ42 compared to those who had a lack of physical activity (p < 0.001). (B) Participants who were physically active had lower p-tau/Aβ42 levels compared to those who had a lack of physical activity (p < 0.001). Supplementary Table 1. Association between lifestyle categories and CSF AD biomarkers; Supplementary Table 2. Association of lifestyle categories with CSF Aβ42/Aβ40, tau/Aβ42 and p-tau/Aβ42 ratios; Supplementary Table 3. Association between lifestyle categories (defined by physical activity and social engagement) and CSF AD biomarkers. Supplementary Table 4. Interaction analysis between physical activity and social engagement. Questionnaire used in the evaluation of healthy lifestyles. [file 13195_2021_822_MOESM1_ESM.pdf]

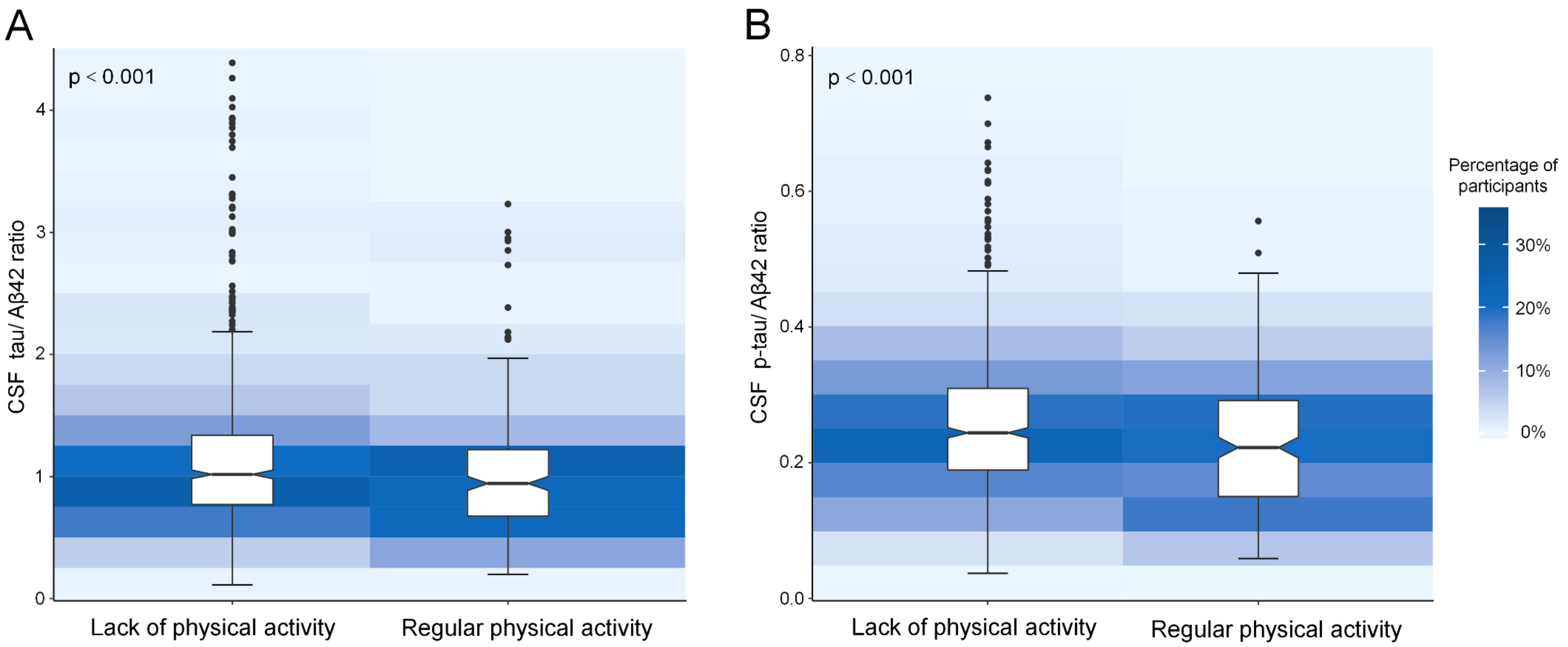

Supplementary Fig.1. Associations of physical activity with CSF tau/Aβ42 and p-tau/ Aβ42 ratios.

(A) Participants who were physically active had lower CSF tau/Aβ42 compared to those who had a lack of physical activity ($p=0.006$). (B) Participants who were physically active had lower p-tau/Aβ42 levels compared to those who had a lack of physical activity ($p < 0.001$).

Supplementary Table 1. Association between lifestyle categories and CSF AD biomarkers

	CSF Aβ42			CSF tau			CSF p-tau		
	β	*p*	*p* for trend	β	*p*	*p* for trend	β	*p*	*p* for trend
All participants			0.39			**<0.01**			**<0.01**
Unfavorable lifestyle	Reference	-		Reference	-		Reference	-	
Intermediate lifestyle	-0.0003	0.10		-0.0048	**0.02**		-0.0055	**0.05**	
Favorable lifestyle	0.0003	0.16		-0.0120	**<0.01**		-0.0150	**<0.01**	
APOE ε4 carriers			0.96			**<0.01**			**0.02**
Unfavorable lifestyle	Reference	-		Reference	-		Reference	-	
Intermediate lifestyle	-0.0004	0.32		-0.0156	**<0.01**		-0.0129	0.06	
Favorable lifestyle	0.0001	0.92		-0.0182	**<0.01**		-0.0214	**0.03**	
APOE ε4 non-carriers			0.48			**<0.01**			**<0.01**
Unfavorable lifestyle	Reference	-		Reference	-		Reference	-	
Intermediate lifestyle	-0.0002	0.18		-0.0026	0.27		-0.0043	0.15	
Favorable lifestyle	0.0004	0.14		-0.0107	**<0.01**		-0.0137	**<0.01**	

Supplementary Table 2. Association of lifestyle categories with CSF Aβ42/Aβ40, tau/Aβ42 and p-tau/Aβ42 ratios

	CSF Aβ42/Aβ40			CSF tau/Aβ42			CSF p-tau/Aβ42		
	β	*p*	*p* for trend	β	*p*	*p* for trend	β	*p*	*p* for trend
All participants			0.99			**<0.01**			**<0.01**
Unfavorable lifestyle	Reference	-		Reference	-		Reference	-	
Intermediate lifestyle	-2.6E-3	0.15		-0.08	0.09		-0.00	0.72	
Favorable lifestyle	1.7E-3	0.52		-0.24	**<0.01**		-0.04	**<0.01**	
APOE ε4 carriers			0.99			**0.02**			0.15
Unfavorable lifestyle	Reference	-		Reference	-		Reference	-	
Intermediate lifestyle	1.1E-3	0.72		-0.23	0.07		-0.01	0.71	
Favorable lifestyle	7.4E-4	0.86		-0.37	**0.04**		-0.05	0.11	
APOE ε4 non-carriers			0.85			**<0.01**			**<0.01**
Unfavorable lifestyle	Reference	-		Reference	-		Reference	-	
Intermediate lifestyle	-3.3E-3	0.15		-0.06	0.24		-0.00	0.89	
Favorable lifestyle	1.2E-3	0.72		-0.29	**<0.01**		-0.04	**<0.01**	

Supplementary Table 3. Association between lifestyle categories (defined by physical activity and social engagement) and CSF AD biomarkers

	CSF Aβ42			CSF tau			CSF p-tau		
	β	*p*	*p* for trend	β	*p*	*p* for trend	β	*p*	*p* for trend
All participants			0.38			**<0.01**			**<0.01**
Unfavorable lifestyle	Reference	-		Reference	-		Reference	-	
Intermediate lifestyle	-0.0002	0.26		-0.0038	0.15		-0.0063	**0.05**	
Favorable lifestyle	0.0002	0.46		-0.0102	**<0.01**		-0.0171	**<0.01**	

Supplementary Table 4. Interaction analysis between physical activity and social engagement.

	CSF Aβ42		CSF tau		CSF p-tau	
	B	*p*	β	*p*	β	*p*
Age	-0.0000	0.87	0.0008	**＜0.01**	0.0007	**＜0.01**
Sex	0.0001	0.34	-0.0009	0.63	-0.0019	0.46
Education	0.0000	0.87	-0.0001	0.74	-0.0004	0.15
APOE	-0.0004	**0.04**	0.0020	0.43	0.0069	**0.03**
Physical activity	0.0004	0.31	0.0019	0.71	0.0052	0.45
Absence of social isolation	-0.0002	0.41	-0.0028	0.31	-0.0045	0.19
Physical activity ×Absence of social isolation	0.0000	0.95	-0.0078	0.17	-0.0152	0.04

Questionnaire used in the evaluation of healthy lifestyles

**Smoking status**	
Do you smoke?	Yes/No
How many cigarettes do you smoke in one day?	
Do you quit smoke?	Yes/No
**Alcohol consumption**	
Do you drink alcohol?	Yes/No
How often do you drink?	times/week
What kind of alcohol do you drink? How much do you drink in one day?	Wine: g/day<br>Liquor: g/day<br>Beer: bottle/day
**Social engagement**	
What is your marital status?	Single/married/separated/divorced/widowed
Do you live alone?	Yes/No
Do you have intimate relationship with your relatives?	Yes/No
Do you have intimate friends who can support you?	Yes/No
Do you have intimate relationship with your neighbors?	Yes/No
**Physical activity**	
Do you have physical exercise regularly?	Yes/No
What kind of physical exercise do you do?	
What is the frequency of your physical exercise?	times/week
